# Use of tramadol and other analgesics following media attention and risk minimization actions from regulators: a Danish nationwide drug utilization study

**DOI:** 10.1007/s00228-020-03016-6

**Published:** 2020-10-28

**Authors:** Anne Mette Skov Sørensen, Lotte Rasmussen, Martin Thomsen Ernst, Stine Hasling Mogensen, Mona Vestergaard Laursen, Espen Jimenez-Solem, Anton Pottegård

**Affiliations:** 1 Department of Clinical Pharmacology, Bispebjerg and Frederiksberg Hospital, Copenhagen, Denmark; 2 Clinical Pharmacology and Pharmacy, Department of Public Health, University of Southern Denmark, JB Winsløwsvej 19, 2, DK-5000 Odense, Denmark; 3 The Danish Medicines Agencys Data Analytics Centre, Copenhagen, Denmark; 4 Copenhagen Phase IV unit (Phase4CPH), Department of Clinical Pharmacology and Center of Clinical Research and Prevention, Bispebjerg and Frederiksberg Hospital, Copenhagen, Denmark; 5 Institute of Clinical Medicine, University of Copenhagen, Copenhagen, Denmark

**Keywords:** Tramadol, Drug utilization, Regulatory action, Opioid use

## Abstract

**Purpose:**

To describe the use of tramadol and other analgesics in Denmark focusing on the impact of media attention (June and December 2017) and regulatory actions (September 2017 and January 2018) on the use of tramadol.

**Methods:**

Using nationwide registries, we identified all adults who filled a prescription for tramadol and other analgesics from 2014 to 2019. We described incidence rates, prevalence proportions, and total use of tramadol and other analgesics over time. We also described switching between analgesics, treatment duration, skewness in drug use, and doctor-shopping.

**Results:**

From early 2017 until the end of 2019, total tramadol use decreased markedly while the use of morphine and oxycodone decreased slightly. The quarterly prevalence of tramadol use decreased from 32/1000 individuals in 2014 to 18/1000 at the end of 2019, dropping mainly at the time of media attention. Concomitantly, the quarterly prevalence increased for oxycodone (from 5.1 to 8.2) and morphine (from 8.5 to 9.8), mainly due to more short-term and sporadic users, and decreased for codeine (14 to 9.6). From 2014 to mid-2017, the incidence of tramadol use was stable (around 2.2/1000 person-months) but dropped in June 2017 to 1.7/1000, coinciding with the media attention. The incidence of tramadol use continued to decrease (to 1.1/1000 at the end of 2019).

**Conclusion:**

We identified a decline in tramadol use coinciding with the media attention in 2017 and continuing during regulatory actions. There was generally no evidence of unintended effects on the utilization of opioids related to the media attention and regulatory actions.

**Electronic supplementary material:**

The online version of this article (10.1007/s00228-020-03016-6) contains supplementary material, which is available to authorized users.

## Introduction

The use of prescription opioids, including tramadol, has increased worldwide during the last decades resulting in a dramatic increase in overdoses and opioid use disorders, especially in the USA [1–3]. In Denmark, the total use of tramadol increased twofold from 2001 to 2013 [4]. Furthermore, from 2006 to 2016, the prevalence of tramadol use was markedly higher in Denmark compared to Sweden and Norway [5], although these countries have rather similar health care systems and cultures.

Tramadol is a centrally acting synthetic opioid with monoaminergic actions, and both abuse and addiction have been reported in several previous studies [6, 7]. Despite this, tramadol has been considered as having a limited potential for abuse and addiction [6, 8, 9] and this matter has in recent years been an issue of growing concern in Denmark [10–12]. In June and December 2017, Danish national television broadcasted two documentaries concerning tramadol and the disputed inadequate information regarding the risk of addiction [10, 11]. Following this, attention from lay media was intense and several pain specialists expressed their concerns over the widespread use of tramadol.

At the same time, there has been an increased awareness by the Danish Health Authority on the increasing use of opioids, including tramadol. Since 2007, several initiatives have been launched including guidelines and educational courses directed at Danish physicians [13, 14]. In addition, from September 2017 and 1 year ahead, the Danish Medicines Agency made it mandatory for physicians to report all suspected adverse drug reactions of tramadol [15]. Furthermore, from January 2018, tramadol and certain other opioids, e.g., codeine, have followed the same regulatory restrictions of prescribing as opioids with a known abuse potential such as morphine and oxycodone. Among others, these restrictions means that tramadol can only be dispensed once per prescription [16].

Previous studies from other countries have observed changes in drug utilization following regulatory actions and media attention [17–19]. Currently, the effects of the Danish regulatory risk minimization actions on the use of tramadol and other analgesics are unknown. Therefore, the aim of this study was to investigate the effects of the regulatory actions by providing detailed analyses of the use of tramadol and other analgesics in Denmark from 2014 to 2019.

## Patients and methods

In this descriptive nationwide drug utilization study, we used the Danish health care registries to describe the use of tramadol and other analgesics including other opioids, nonsteroidal anti-inflammatory drugs (NSAIDs), gabapentinoids, tricyclic antidepressants (TCAs), and serotonin and norepinephrine reuptake inhibitors (SNRIs) in adults from 2014 to 2019.

### Data sources

The Danish National Prescription Registry contains individual-level information on all prescription drugs dispensed at Danish outpatient pharmacies since 1995 [20]. Prescription data includes the personal identification number, date of dispensing, quantity, strength, type of drug, and a prescriber identifier. Drugs are coded according to the Anatomical Therapeutic Chemical classification (ATC) system and the quantity in each prescription is expressed by the Defined Daily Dose (DDD) [21]. By linking the prescriber identifier with information in the Registry of Health Care Providers [22], we were able to identify the medical specialty of prescribers from the primary sector. The information on prescribers in the Danish National Prescription Registry has previously been found to be valid [23].

Data on hospital diagnoses was obtained from the Danish National Patient Register [24] and we used the Danish Civil Registration System to obtain information on birth, death, and migrations [25].

The data sources were linked by a unique identifier (CPR-number), which is assigned to all Danish residents at birth or immigration.

All drug (ATC codes) and diagnosis codes (International Classification of Diseases (ICD-10 codes)) used in this study are provided in Online resource 1.

### Study cohort and study drugs

The study cohort included all adults (≥ 18 years) living in Denmark from January 1, 2014, to December 31, 2019. For this cohort, we identified all filled prescriptions for tramadol, other opioids, NSAIDs, gabapentinoids (gabapentin and pregabalin), TCAs, and SNRIs. We included the use of NSAIDs and gabapentinoids, to assess possible changes in utilization patterns of other non-opioid analgesics, and TCAs and SNRIs to assess switching between analgesics. We differentiated between new users and prevalent users. New users were defined as individuals redeeming a prescription with no prior prescription for the same drug or drug class within the last 5 years.

Opioids included all opioids available for oral or transdermal use (excluding intravenous formulations used in out-patient settings, suppositories, nasal sprays etc., all of which are very rarely used in Denmark). We categorized opioids according to the four most frequently used individual opioids in Denmark: morphine, oxycodone, tramadol, and codeine. We included a category with “other opioids,” which included the remaining opioids in the ATC group for opioids (N02A*), which, when considering volumes sold, mainly comprised transdermal opioids. In Denmark, three combination products of codeine are available, and these were all included in the codeine category. In order to restrict the analysis to drugs used to treat pain, we only included codeine tablets (i.e., disregarding oral suspensions used in the treatment of cough).

We used oral morphine equivalents (OMEQs) to quantify the equipotent amount of opioids used and applied conversion factors recently published by Svendsen et al. and Jarlbaek [26, 27]. Conversion factors for the study drugs are provided in Online resource 1. Often, consumption of drugs is measured using the DDD, which is a predefined international standard amount of adult daily dose assigned by the WHO (World Health Organization) [21]. However, the assigned DDD for opioids does not reflect the potency of the different opioids. To enable comparison with previous studies, we therefore, in supplementary analyses, also used DDDs to quantify the amount of opioids used.

Time of media attention was determined by the first documentary broadcasted in June 2017 and supported by a Google Trends query showing a large search activity of “Tramadol” in Denmark during June 2017 [10, 28].

### Analyses

We divided the analysis into seven parts where we described the use of tramadol and other analgesics during 2014–2019 and described changes in treatment patterns over time by comparing three study years, i.e., 2016 (before media attention and regulatory actions), 2017 (during media attention and regulatory actions), and 2019 (after media attention and regulatory actions).

First, we described users of tramadol and other opioids according to sex, age, and morbidities. This was done separately for users in 2016, 2017, and 2019. Morbidities were assessed as hospital diagnoses recorded in the Patient Registry at any time prior to January 1 of the year being analyzed.

Second, three separate analyses were performed to describe changes in analgesic use over time from January 1, 2014, to December 31, 2019. These included (A) total drug use per month, calculated by dividing the amount of dispensed milligram OMEQs (or DDDs in the supplementary analyses) by the number of adults living in Denmark on the 1st day in each quarter; (B) yearly and quarterly prevalence proportions of use of tramadol, other opioids, NSAIDs, and gabapentinoids, calculated for all adults living in Denmark on January 1 for each year or, for the quarterly prevalence, 1st day in each quarter in the denominator; and finally, (C) monthly incidence rates calculated by dividing the monthly number of new users (as defined above) by the total number of adults in Denmark on the 1st day in each quarter.

Third, to assess possible switches between analgesics in relation to the use of tramadol, we calculated the proportion of new tramadol users in 2016, 2017, and 2019 who had filled other analgesics (NSAIDs, gabapentinoids, opioids, TCAs, or SNRIs) during the previous 3 months or during the 3 months according to the first tramadol prescription.

Fourth, as a crude measure of treatment duration, we calculated the proportion of new tramadol users who redeemed 1, 2, or ≥ 3 tramadol prescriptions in the year following their first tramadol prescription. The analysis was performed separately for 2016, 2017, and 2018 (to ensure a full year of follow-up). The crude measure of duration was applied due to preliminary analyses showing a very low number of long-term users, rendering “drug survival” analyses less useful [29].

Fifth, we investigated the medical specialty of prescribers issuing prescriptions for tramadol. We calculated the proportion of incident tramadol prescriptions and prevalent tramadol prescriptions (defined as non-incident) issued by different types of prescribers in 2016, 2017, 2018, and 2019. The type of prescriber was classified into six categories including (1) general practitioners (GPs) (including on-call GPs), (2) private practicing specialists, (3) hospital prescribers, (4) dentists, (5) others, and 6) missing.

Sixth, we described the skewness of tramadol use by generating Lorenz curves for the years 2016, 2017, and 2019. Lorenz curves describe skewness in the use of a drug on a population level and have been described in detail elsewhere [30]. It may be used as a marker of potential abuse and sporadic drug use. The Gini coefficient, a value ranging from 0 to 1 with 0 denoting total equality and 1 denoting full inequality of use, and the 1st, 10th, 50th, and 90th percentiles were calculated for each Lorenz curve.

Finally, to assess the occurrence of doctor shopping among users of tramadol, codeine, oxycodone, and morphine, we calculated the proportion of individuals with 1, 2, 3, 4, or ≥ 5 different unique prescribers in 2016, 2017, 2018, or 2019. To ensure a relevant population, this analysis was restricted to individuals redeeming ≥ 5 prescriptions of the given drug during the given year. In Denmark, 98% of the population are assigned to a specific GP practice which acts as a gate-keeper to specialist treatment [31]. The number of unique prescribers was therefore considered relevant to reflect doctor-shopping.

All analyses and graphics were performed using Stata Release 15.2 (StataCorp, College Station, Texas, USA).

### Ethics and data protection

Approval from the Ethics Committee was not required according to Danish law [32]. In terms of data protection, the study was registered at the University of Southern Denmark’s inventory (record no. 10.490).

## Results

We identified 1,328,568 unique users of opioids of which 63% had redeemed one or more prescription for tramadol between January 1, 2014, and December 31, 2019. In 2019, 247,335 (59%) of opioid users were female and the median age was 63 (IQR, 49–75) years (Table 1). Users of tramadol and codeine were slightly younger compared to users of the remaining opioids. There were only limited variations in history of morbidities between users of different opioids. However, users of tramadol and codeine generally had fewer comorbidities compared to users of morphine and oxycodone, and in particular compared to those using “other opioids” (mainly comprising transdermal opioid formulations). Furthermore, users of morphine more commonly had a history of cancer. Corresponding tables for 2016 and 2017 are presented in Online resource 2, Tables 1 and 2.

**Table 1:** Characteristics of opioid users in 2019 overall and specified by type of opioid

	Any opioid	Morphine	Oxycodone	Tramadol	Codeine	Other opioids
	(*n* = 422,346)	(*n* = 98,803)	(*n* = 87,302)	(*n* = 185,087)	(*n* = 105,754)	(*n* = 34,838)
Female sex (%)	247,335 (58.6%)	54,536 (55.2%)	50,125 (57.4%)	105,791 (57.2%)	69,527 (65.7%)	22,801 (65.4%)
Age (years)^1^	63 (49–75)	66 (52–76)	66 (52–76)	61 (48–73)	62 (48–73)	75 (62–85)
Essential hypertension	112,499 (26.6%)	29,196 (29.5%)	26,453 (30.3%)	49,159 (26.6%)	24,407 (23.1%)	14,154 (40.6%)
Ischemic heart disease	57,893 (13.7%)	15,744 (15.9%)	12,967 (14.9%)	25,763 (13.9%)	12,444 (11.8%)	6807 (19.5%)
Chronic kidney disease	10,060 (2.4%)	2397 (2.4%)	2856 (3.3%)	4592 (2.5%)	1675 (1.6%)	1563 (4.5%)
Osteoarthritis	110,067 (26.1%)	28,263 (28.6%)	30,115 (34.5%)	47,390 (25.6%)	22,960 (21.7%)	11,149 (32.0%)
Diabetes	61,429 (14.5%)	15,872 (16.1%)	13,279 (15.2%)	28,026 (15.1%)	13,669 (12.9%)	6472 (18.6%)
Affective disorders	39,334 (9.3%)	10,415 (10.5%)	8528 (9.8%)	17,898 (9.7%)	8436 (8.0%)	4779 (13.7%)
Depression	36,283 (8.6%)	9648 (9.8%)	7938 (9.1%)	16,467 (8.9%)	7705 (7.3%)	4487 (12.9%)
Migraine	10,928 (2.6%)	2254 (2.3%)	2086 (2.4%)	4838 (2.6%)	3519 (3.3%)	807 (2.3%)
Cancer^2^	55,560 (13.2%)	19,628 (19.9%)	13,852 (15.9%)	20,514 (11.1%)	11,124 (10.5%)	8588 (24.7%)
Substance abuse^3^	40,906 (9.7%)	11,494 (11.6%)	8996 (10.3%)	19,208 (10.4%)	7975 (7.5%)	4260 (12.2%)
COPD	35,228 (8.3%)	11,507 (11.6%)	8050 (9.2%)	15,010 (8.1%)	7152 (6.8%)	4637 (13.3%)
Pain	61,403 (14.5%)	18,037 (18.3%)	15,628 (17.9%)	25,250 (13.6%)	10,299 (9.7%)	10,184 (29.2%)
Acute	5146 (1.2%)	1509 (1.5%)	1254 (1.4%)	2233 (1.2%)	984 (0.9%)	709 (2.0%)
Chronic	37,288 (8.8%)	11,466 (11.6%)	9672 (11.1%)	14,823 (8.0%)	5398 (5.1%)	6994 (20.1%)
Fibromyalgia	3289 (0.8%)	749 (0.8%)	653 (0.7%)	1664 (0.9%)	746 (0.7%)	370 (1.1%)
Rheumatoid arthritis	6741 (1.6%)	1776 (1.8%)	1578 (1.8%)	3122 (1.7%)	1402 (1.3%)	795 (2.3%)

^1^Results are presented as median (IQR)

^2^Cancers excluding non-melanoma skin cancer

^3^Mental and behavioral disorders due to psychoactive substance use

*COPD*, chronic obstructive pulmonary disease

**Fig. 1:**
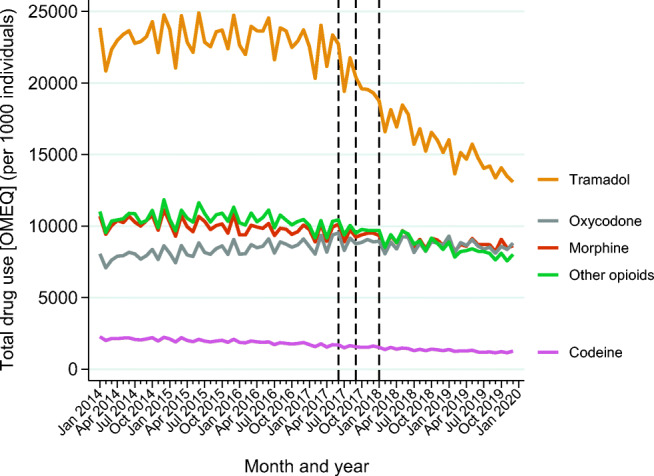
Total use of opioids measured in milligram OMEQ per 1000 individuals and specified by different opioids per month from 2014 to 2019. The three dashed vertical lines denote the time of the media attention (June 2017) and regulatory actions (September 2017 and January 2018) (see the main text for additional details)

From May 2017, just before the first documentary was aired, and until the end of 2019, the total opioid consumption decreased (from 54,690 to 39,767 mg OMEQ per month/1000 individuals) and the total tramadol use decreased (from 23,352 to 13,091 mg OMEQ per month/1000 individuals). Concomitantly, the total use of oxycodone (from 9377 to 8793 OMEQ per month/1000 individuals) and morphine (from 9922 to 8618 OMEQ per month/1000 individuals) decreased slightly (Fig. 1). The total use measured in DDDs per month/1000 individuals is provided in Online resource 2, Fig. 1.

**Fig. 2:**
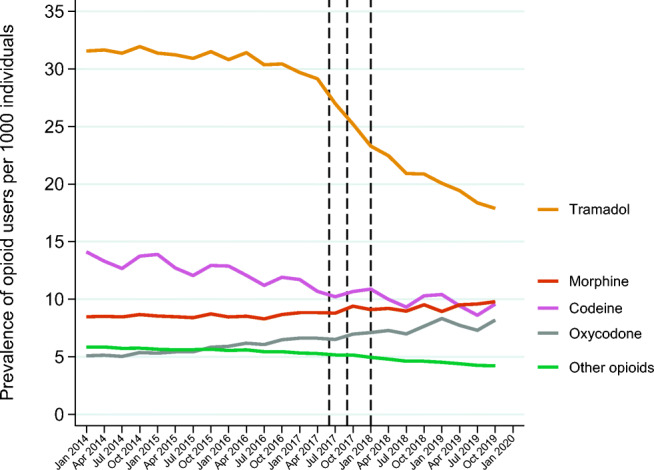
Prevalence proportion of opioid users per 1000 individuals per quarter from 2014 to mid-2019. Vertical lines represent media attention (June 2017), stricter reporting requirement (September 2017), and reclassification (January 2018) (see the main text for additional details)

From 2014 to the end of 2019, we observed a decrease in the prevalence of tramadol use from 32/1000 individuals in the first quarter of 2014 to 18/1000 individuals in the last quarter of 2019 (Fig. 2). Before the media attention, a slightly decreasing trend was noted; however, the decline accelerated at the time of media attention and persisted throughout and after the regulatory initiatives (Fig. 2). From 2014 to the end of 2019, the prevalence increased for oxycodone use (from 5.1 to 8.2/1000 individuals) and morphine use (from 8.5 to 9.8/1000 individuals), while it decreased for codeine (from 14 to 9.6/1000 individuals). Yearly prevalence proportions are presented in Online resource 2, Fig. 2. The prevalence of gabapentinoid use increased from 11 to 18/1000 individuals from 2014 to the end of 2019 while the prevalence of prescribed NSAID use decreased from 65 to 55/1000 individuals (Online resource 2, Fig. 3).

**Fig. 3:**
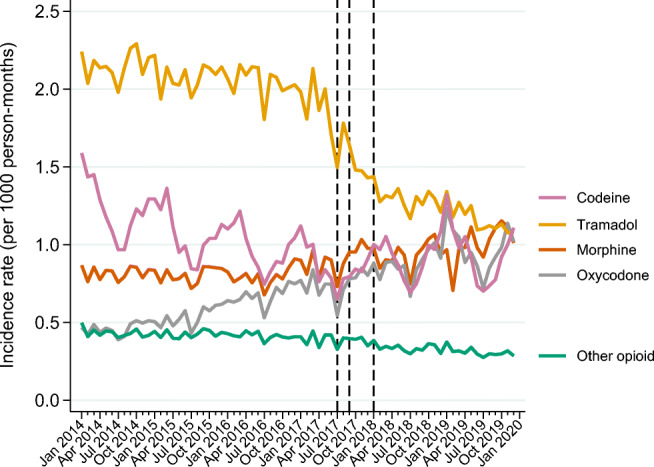
Monthly incidence rate of opioid users between 2014 and 2019. Vertical lines represent media attention (June 2017), stricter reporting requirement (September 2017), and reclassification (January 2018). Monthly incidence rates are calculated by dividing the monthly number of new users by the total number of adults in Denmark on the 1st day in each quarter

In the beginning of the study period, the incidence rate of tramadol use was reasonably stable (approximately 2.2/1000 person-months) but dropped in June 2017 to 1.7/1000 person-months coinciding with the media attention and further to 1.6/1000 person-months and 1.4/1000 person-months in September 2017 and January 2018, respectively (Fig. 3). At the end of 2019, the incidence of tramadol use was 1.1/1000 person-months. From 2014 to the end of 2019, the incidence rate for morphine use (from 0.87 to 1.01/1000 person-months) and oxycodone use increased (from 0.47 to 1.02/1000 person-months) and decreased for codeine (from 1.6 to 1.11/1000 person-months) (Fig. 3). Both the prevalence and incidence rate of codeine exhibited a periodical rhythm.

The proportion of new tramadol users in 2019 who had filled a prescription for NSAIDs, TCAs, SNRIs, gabapentinoids, or another opioid within 3 months before initiating tramadol was 22%, 0.89%, 1.8%, 3.8%, and 7.9%, respectively, while the proportion of new tramadol users who in the subsequent 3 months filled a prescription for these drugs were 34%, 1.5%, 2.0%, 8.4%, and 16%, respectively. The same patterns were observed in the years 2016, 2017, and 2018 (data not shown). An exploratory post hoc analysis revealed that in 2014, 33% of individuals who initiated morphine or oxycodone had used tramadol in the 3 months leading up to them initiating morphine/oxycodone. In 2019, this proportion was reduced to 16%.

The proportion of new tramadol users who filled only one tramadol prescription increased from 63% in 2016 to 73% in 2018. Furthermore, the proportion who filled 2 or ≥ 3 prescriptions in 2016 was 17% and 21%, respectively, and the corresponding numbers were 15% and 12% in 2018, respectively.

Tramadol was primarily initiated by GPs (in 2019: 62%) and hospital prescribers (in 2019: 26%). This changed slightly over time from 2016 to 2019, with a slight decrease in the proportion of new treatments initiated by GPs (from 68 to 62%). Considering prevalent prescriptions, tramadol treatment was mainly maintained by GPs (in 2019: 92%) with no variation over time (Online resource 2, Tables 3 and 4).

**Fig. 4:**
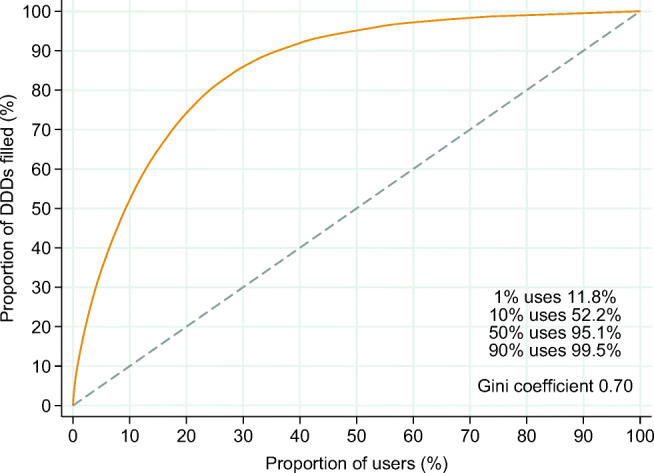
Lorenz curve of tramadol use in 2019. The curve denotes the proportion *X* of the total filled amount of tramadol (measured in defined daily doses (DDDs)) in 2019 that is accounted for by the proportion *X* of users

There was a skewed distribution of tramadol use in 2019 with a Gini coefficient of 0.70, a 1st percentile of 11.8%, and a 50th percentile of 95.1% indicating a small proportion of heavy or long-term users and a high proportion of sporadic or short-term users (Fig. 4). The Lorenz curves for 2016 and 2017 showed similar distributions of tramadol use (Online resource 2, Fig. 4 and 5).

When restricting to patients with ≥ 5 prescriptions for tramadol in a given year, the majority had their prescriptions from only one (in 2019: 72%) or two prescribers (in 2019: 22%). The proportion of tramadol users who had tramadol prescribed by ≥ 5 prescribers declined from 0.74% in 2016 to 0.29% in 2019. Corresponding results are available for other opioids in Online resource 2, Table 5. As an example, 1.1% of morphine and 1.4% of oxycodone users with ≥ 5 prescriptions had their prescriptions prescribed by ≥ 5 prescribers in 2019.

## Discussion

In this nationwide study of the use of tramadol and other analgesics, we found a considerable decrease in both incidence, prevalence, and total use of tramadol coinciding with the media attention in June 2017, a decline which persisted during the regulatory actions. In the same period, a slight increase was noted in the number of new users of morphine and oxycodone, however, with a slight decline in the total use of these drugs, indicating a larger proportion of short-term or sporadic users. Further, the use of gabapentinoids increased and that of NSAIDs decreased during the study period. Tramadol was mainly initiated by GPs as well as hospital prescribers, while continued use was almost exclusively prescribed by GPs. The proportion of heavy or long-term tramadol users was low, while sporadic or short-term tramadol use was more common, which warrants further investigation. However, the skewed distribution of tramadol use was consistent over time, i.e., seemingly not a result of media attention or regulatory actions. There was very limited and diminishing evidence of doctor-shopping for tramadol. We identified a periodical rhythm in both the prevalence and incidence of codeine, which might reflect use of codeine tablets as cough suppressants.

The principle strength of the study is the use of nationwide registries of high quality [20, 24, 25], which allows full capture of all adults in Denmark and their use of opioids and non-opioid analgesics with no risk of recall bias. The main limitation is that the prescription registry does not contain information of over-the-counter (OTC) drugs and drug use during hospital stays. In Denmark, only few analgesics are however available OTC (paracetamol, drugs combining codeine and acetylsalicylic acid, and low-dose ibuprofen in small packages of maximum 20 tablets) and the use of these drugs are consequently not fully captured in the registry. As an example, in 2017, 23% of the total sales of ibuprofen in Denmark was OTC [33]. Furthermore, information on the indication for treatment was not available and it is likely that in particular gabapentinoids and antidepressants were prescribed for other indications than pain. Lastly, we do not know whether the drugs were actually taken by the patients and we only had data on diagnoses from hospitals.

Media attention affecting use of drug therapy has been described numerous times. An example is the debate on the benefit:risk ratio of statins following publications of several articles in The BMJ [34, 35], where it was subsequently shown that patients were more likely to stop their treatment during the debate [19]. Another prominent example from Denmark was the negative public attention and massive media coverage of the alleged side effects of the human papillomavirus vaccine, leading to a dramatic decline in vaccination uptake [36]. This example reflects the great impact also identified in this study and underlines the responsibility held by lay media. While some initiatives targeting opioid prescribing patterns were already initiated by the Danish Health Authority prior to media attention, our results clearly highlight the immediate impact of the news attention to tramadol in 2017. This decline continued during the concomitant regulatory actions from the Danish Medicine Agency in September 2017 and January 2018. Regulatory actions have previously been shown to be effective in addressing use of tramadol in the UK [17] and reduce opioid use and initial prescribing decisions in Florida [37]. However, restrictions in opioid prescribing for acute pain in the USA have also shown disappointing early results and it is possible that some restrictions reduce opioid use more than others [38]. Importantly, direct causality cannot be inferred from our data, that is, it is, e.g., unknown to what extent the continued decline would have been seen regardless of regulatory actions and the continued information on appropriate use of opioids by the Danish Health Authority. However, considering the marked decline in both the total number of users and the rate of new users of tramadol along with the marked decrease in total use, we consider it unlikely that the prolonged decline can be attributed solely to the media attention in 2017.

Interruptions in prescribing patterns, e.g., due to media attention or regulatory actions, can have unforeseen and unwanted consequences. As an example, Chen et al. found an increase in the use of selective serotonin reuptake inhibitors (SSRIs), benzodiazepines (BZDs), and SNRIs after the reclassification of tramadol in the UK [39]. While our data provide an indication that morphine is used instead of tramadol for some patients, as judged by the slight increase in new users and the increase in sporadic or short-term users, the overall use of other opioids is decreasing and there is even a decline in the proportion of users switching to morphine or oxycodone from tramadol. There is, however, some increase in the use of gabapentinoids. Whether this is a downstream consequence of the changes to tramadol use and, if so, whether this represents rational or irrational use of gabapentinoids is currently unknown. However, recent studies have stressed the risk of abuse with gabapentinoids and thus continuous monitoring of the use of gabapentinoids is of paramount importance [40, 41]. Further, how the use of non-pharmacological interventions has changed during this period and whether the new levels of opioid use seen toward the end of the study period represents an optimal level of opioid use is also unknown. Continued efforts should be provided into monitoring the use of opioids in Denmark.

## Conclusion

We identified a decline in the use of tramadol coinciding in time with the media attention in 2017. This decline continued during the regulatory risk minimization actions taken in 2017–2018. During the same period, the use of other opioids decreased slightly. There was generally no evidence of unintended effects on the utilization of opioids related to the media attention and regulatory actions. In total, this provides strong evidence for the combined effect of media attention and regulatory actions in changing prescribing practices within a therapeutic area which is currently much debated.

## Electronic supplementary material

ESM 1 (DOCX 25 kb)

ESM 2 (DOCX 329 kb)
