# Supplementary material for: Use of tramadol and other analgesics following media attention and risk minimization actions from regulators: a Danish nationwide drug utilization study
Source: Eur J Clin Pharmacol. 2020 Oct 28;77(4):617–24. doi: 10.1007/s00228-020-03016-6 (PMC7935826; doi:10.1007/s00228-020-03016-6)
Supplement: Supplementary file 1 — (DOCX 25 kb) [file 228_2020_3016_MOESM1_ESM.docx]

**Use of tramadol and other analgesics
following media attention and risk minimization actions from regulators:
A Danish nationwide drug utilization study**

**European Journal of Clinical Pharmacology**

**Online Resource 1**

Authors: Anne Mette Skov Sørensen, Lotte Rasmussen, Martin Thomsen Ernst, Stine Hasling Mogensen, Mona Vestergaard, Espen Jimenez Solem and Anton Pottegård

Corresponding author: Anton Pottegård, Clinical Pharmacology, University of Southern Denmark, E-mail: [apottegaard@health.sdu.dk](mailto:apottegaard@health.sdu.dk)

**Contents**

Codes and definitions

Table 1. Anatomical Therapeutic Chemical classification (ATC) and International Classification of Diseases (ICD10) codes

Table 2. Defined Daily Doses (DDDs) and conversion factors

Our population included all Danes redeeming a prescription for NSAIDs (M01A*), opioids (N02A*, R05DA04 and N02BA75), gabapentin or pregabalin (N03AX12 and N03AX16, respectively) from January 1, 2014 to December 31, 2018. ATC-groups and ICD10 codes are available in Table 1. DDDs and conversion factors are available in Table 2. For reference, DDDs are available from the World Health Organization (WHO). ‘Other opioids’ included the following ATC codes: N02AA03 ([hydromorphon](https://pro.medicin.dk/Medicin/Indholdsstoffer/2278)e), N02AA04 (nicomorphine), N02AA55 (oxycodone and naloxone), N02AB02 (pethidine), N02AB03 (fentanyl), N02AE01 (buprenorphine), N02AF02 (nalbuphine), N02AG02 ([ketobemidon](https://pro.medicin.dk/Medicin/Indholdsstoffer/602)e and antispasmodics), N02AX06 (tapentadol).

| **Table 1** ATC and ICD10 codes | |
| --- | --- |
| *Drug* | *ATC code* |
| Opioids | N02A |
| NSAID | M01A |
| Morphine | N02AA01 |
| Codeine* | R05DA04, N02AJ06, N02AJ07, N02BA75 |
| Oxycodone | N02AA05 |
| Tramadol | N02AX02 |
| *Gabapentinoids* |  |
| - Gabapentin | N03AX12 |
| - Pregabalin | N03AX16 |
| *SNRI* |  |
| - Venlafaxine | N06AX16 |
| - Duloxetine | N05AX21 |
| *TCA* |  |
| - Amitriptyline | N06AA09 |
| - Nortriptyline | N06AA10 |
| - Imipramine | N06AA02 |
| *Comorbidity* | *ICD-10 code* |
| Essential hypertension | [I10](http://www.diagnosekoder.dk/#/search/DI109) |
| Ischemic heart disease | I20-25 |
| Chronic kidney disease | N18, N182, N183, N184, N185, N181, N189, N118 |
| Osteoarthritis | M15-M19 |
| Diabetes | E10-E14, (prescriptions: A10) |
| Affective disorders including depression | F30-39 |
| Depression | F32-F33 |
| Migraine | G43 |
| Cancer (excluding nonmelanoma skin cancer) | C00-C98 (excluding C44) |
| Mental and behavioral disorders due to psychoactive substance use | F10-19 |
| Chronic obstructive pulmonary disease | J42-44 |
| Pain  Acute  Chronic | R52, R529  R520  R522A, R522 |
| Fibromyalgia | M797 |
| Rheumatoid arthritis | M05 |
| *When analyzing codeine, we included the three codeine combinations available in Denmark (N02BA75, N02AJ06 and N02AJ07) | |

| **Table 2** DDDs and conversion factors | | | |
| --- | --- | --- | --- |
| *Drug* | *Mg/DDD* | *Equianalgesic potency in relation to oral morphine** | *Conversion factor^**^* |
| Morphine | 100 | 1 | 100 |
| Codeine | 100 | 0.1 | 10 |
| Oxycodone | 75 | 1.5 | 112.5 |
| Tramadol | 300 | 0.2 | 60 |
| [Hydromorphon](https://pro.medicin.dk/Medicin/Indholdsstoffer/2278)e | 4 | 7.5 | 30 |
| Nicomorphine | 30 | 1 | 30 |
| Oxycodone and naloxone | 75 | 1.5 | 112.5 |
| Pethidine | 400 | 0.1 | 40 |
| Fentanyl (transdermal) | 1.2 | 100 | 120 |
| Fentanyl (sublingual) | 0.6 | 50 | 30 |
| Buprenorphine (sublingual) | 1.2 | 75 | 90 |
| Buprenorphine (transdermal) | 1.2 | 110 | 132 |
| K[etobemidon](https://pro.medicin.dk/Medicin/Indholdsstoffer/602)e and antispasmodics | 25 | 1 | 25 |
| Tapentadol | 400 | 0.33 | 132 |
| Ketobemidone | 50 | 1 | 50 |
| Dextropropoxyphene | 300 | 0.15 | 45 |
| Pentazocine | 200 | 0.17 | 34 |
| *Equianalgesic potencies between oral morphine and oral opioids, except for fentanyl and buprenorphine, where the potencies refer to transdermal route and sublingual route  **The conversion factors are the factors used to estimate the amounts of mg oral morphine equivalents by multiplication with the amounts of DDDs | | | |
