# Supplementary material for: Use of tramadol and other analgesics following media attention and risk minimization actions from regulators: a Danish nationwide drug utilization study
Source: Eur J Clin Pharmacol. 2020 Oct 28;77(4):617–24. doi: 10.1007/s00228-020-03016-6 (PMC7935826; doi:10.1007/s00228-020-03016-6)
Supplement: Supplementary file 2 — (DOCX 329 kb) [file 228_2020_3016_MOESM2_ESM.docx]

**Use of tramadol and other analgesics
following media attention and risk minimization actions from regulators:
A Danish nationwide drug utilization study**

**European Journal of Clinical Pharmacology**

**Online resource 2**

Authors: Anne Mette Skov Sørensen, Lotte Rasmussen, Martin Thomsen Ernst, Stine Hasling Mogensen, Mona Vestergaard, Espen Jimenez Solem and Anton Pottegård

Corresponding author: Anton Pottegård, Clinical Pharmacology, University of Southern Denmark, E-mail: [apottegaard@health.sdu.dk](mailto:apottegaard@health.sdu.dk)

**Contents**

Figure 1. Total use of opioids per 1000 individuals measured in Defined Daily Doses (DDD) and specified by different opioids per month from 2014 to 2019

Figure 2. Prevalence proportion per 1000 individuals per year from 2014 to 2019, opioids

Figure 3. Prevalence proportion per 1000 individuals per quarter from 2014 to 2019, nonsteroidal anti-inflammatory drugs (NSAIDs) and gabapentinoids

Figure 4. Lorenz curves for tramadol 2016

Figure 5. Lorenz curves for tramadol 2017

Table 1. Gender-, age- and morbidity-characteristics for opioid users in 2016

Table 2. Gender-, age- and morbidity-characteristics for opioid users in 2017

Table 3. Medical profile of prescribers issuing prescriptions of tramadol among prevalent tramadol users

Table 4. Medical profile of prescribers issuing prescriptions of tramadol among new tramadol users

Table 5. Number of unique prescribers among opioid users during the given year

**Figure 1. Total monthly use of opioids per 1000 individuals measured in defined daily doses (DDD) and specified by different opioids from 2014 to 2019. The three dashed vertical lines denote the time of the media attention (June 2017) and regulatory actions (September 2017 and January 2018) – see the main text for additional details.**


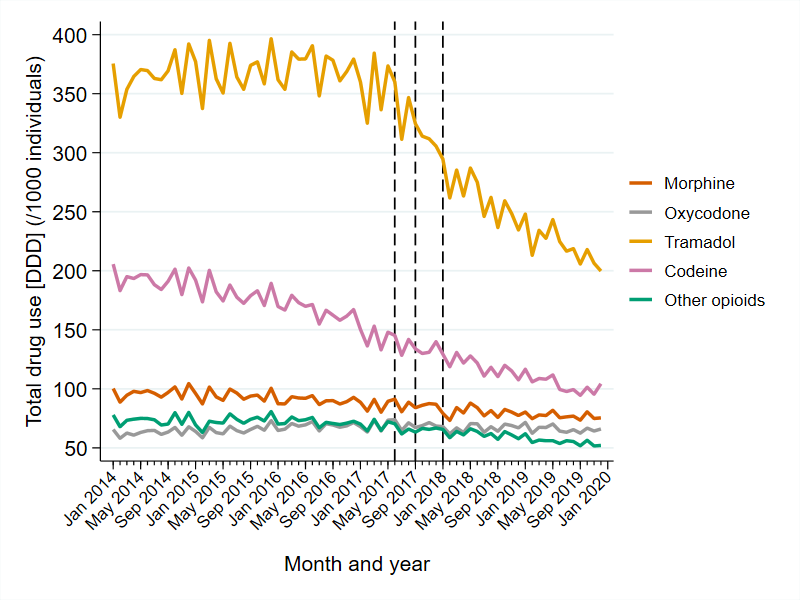


**Figure 2. Annual prevalence proportion per 1000 individuals of opioid users from 2014 to 2019.**


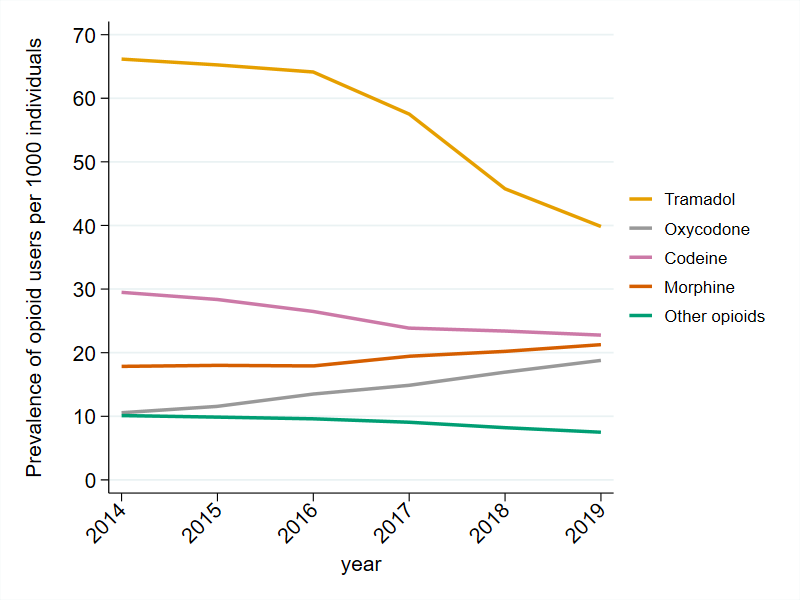


**Figure 3. Quarterly prevalence proportion per 1000 individuals of nonsteroidal anti-inflammatory drug (NSAID) and gabapentinoid use from 2014 to 2019. The three dashed vertical lines denote the time of the media attention (June 2017) and regulatory actions (September 2017 and January 2018) – see the main text for additional details.**


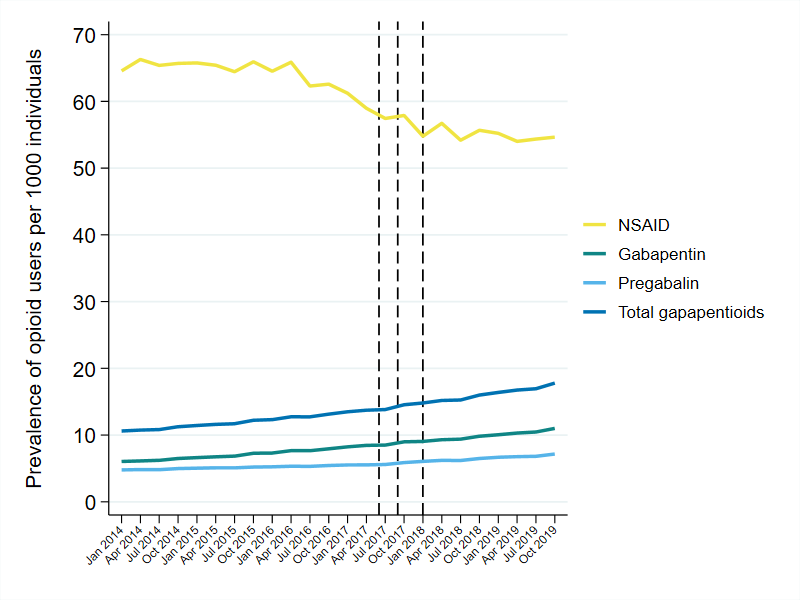


**Figure 4. Lorenz curves of tramadol use in 2016.** **The curve denotes the proportion X of the total filled amount of tramadol (measured in defined daily doses (DDDs)) in 2016 that is accounted for by the proportion X of users.**


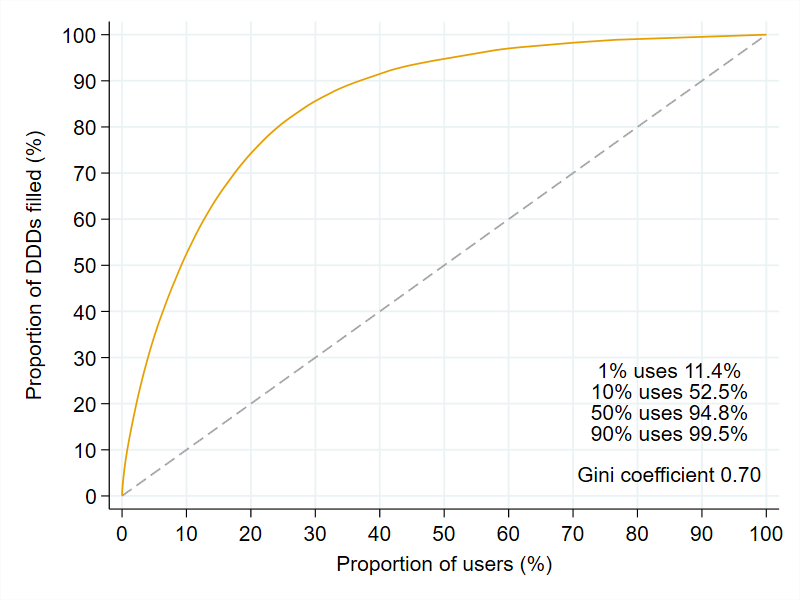


**Figure 5**. **Lorenz curves of tramadol use in 2017. The curve denotes the proportion X of the total filled amount of tramadol (measured in defined daily doses (DDDs)) in 2017 that is accounted for by the proportion X of users.**


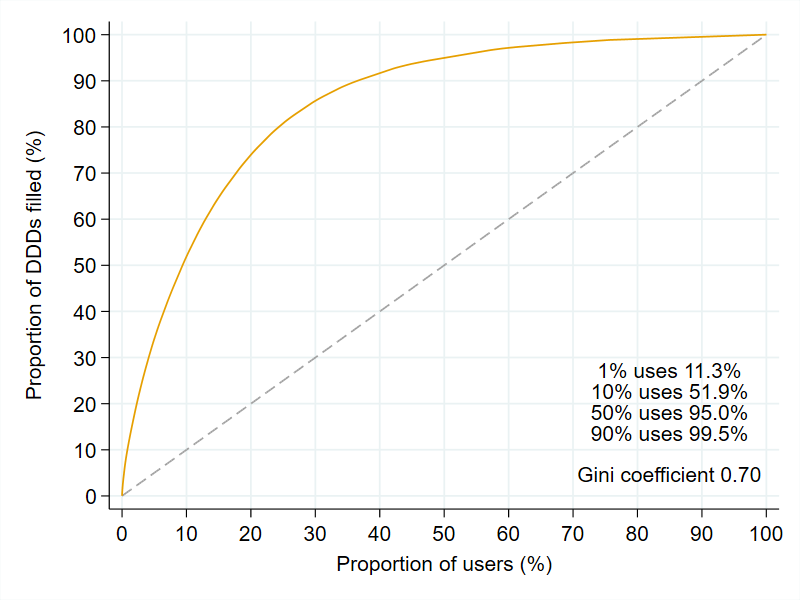


**Table 1 Gender-, age- and morbidity-characteristics for opioid users in 2016**

|  | Any opioid | Morphine | Oxycodone | Tramadol | Codeine | Other opioids |
| --- | --- | --- | --- | --- | --- | --- |
|  | (n=487,239) | (n=81,375) | (n=61,309) | (n=291,149) | (n=120,243) | (n=43,627) |
| Female sex (%) | 286,178 (58.7%) | 44,753 (55.0%) | 34,888 (56.9%) | 166,868 (57.3%) | 79,640 (66.2%) | 28,353 (65.0%) |
| Age (years)^1^ | 61 (47-73) | 65 (52-76) | 64 (50-75) | 60 (46-72) | 60 (47-72) | 74 (60-84) |
| Essential hypertension | 117,268 (24.1%) | 23,349 (28.7%) | 17,284 (28.2%) | 69,603 (23.9%) | 25,390 (21.1%) | 16,257 (37.3%) |
| Ischemic heart disease | 63,963 (13.1%) | 12,935 (15.9%) | 9,313 (15.2%) | 38,305 (13.2%) | 13,682 (11.4%) | 8,592 (19.7%) |
| Chronic kidney disease | 10,042 (2.1%) | 1,803 (2.2%) | 1,981 (3.2%) | 6,184 (2.1%) | 1,694 (1.4%) | 1,782 (4.1%) |
| Osteoarthritis | 109,234 (22.4%) | 21,715 (26.7%) | 20,081 (32.8%) | 64,872 (22.3%) | 22,861 (19.0%) | 12,611 (28.9%) |
| Diabetes | 65,769 (13.5%) | 12,700 (15.6%) | 9,222 (15.0%) | 39,878 (13.7%) | 14,667 (12.2%) | 7,480 (17.1%) |
| Affective disorders | 42,612 (8.7%) | 8,636 (10.6%) | 5,942 (9.7%) | 25,496 (8.8%) | 9,353 (7.8%) | 5,704 (13.1%) |
| Depression | 39,192 (8.0%) | 7,984 (9.8%) | 5,517 (9.0%) | 23,412 (8.0%) | 8,493 (7.1%) | 5,319 (12.2%) |
| Migraine | 10,183 (2.1%) | 1,596 (2.0%) | 1,331 (2.2%) | 5,860 (2.0%) | 3,254 (2.7%) | 859 (2.0%) |
| Cancer^2^ | 54,340 (11.2%) | 15,886 (19.5%) | 9,285 (15.1%) | 28,452 (9.8%) | 10,939 (9.1%) | 9,687 (22.2%) |
| Substance abuse^3^ | 44,908 (9.2%) | 10,019 (12.3%) | 6,259 (10.2%) | 27,496 (9.4%) | 8,940 (7.4%) | 4,858 (11.1%) |
| COPD | 37,304 (7.7%) | 9,309 (11.4%) | 5,488 (9.0%) | 21,822 (7.5%) | 7,748 (6.4%) | 5,672 (13.0%) |
| Pain | 53,561 (11.0%) | 13,910 (17.1%) | 10,725 (17.5%) | 27,893 (9.6%) | 8,956 (7.4%) | 10,341 (23.7%) |
| Acute | 5,029 (1.0%) | 1,251 (1.5%) | 947 (1.5%) | 2,849 (1.0%) | 957 (0.8%) | 759 (1.7%) |
| Chronic | 32,195 (6.6%) | 8,886 (10.9%) | 6,867 (11.2%) | 16,012 (5.5%) | 4,808 (4.0%) | 6,854 (15.7%) |
| Fibromyalgia | 2,723 (0.6%) | 486 (0.6%) | 365 (0.6%) | 1,669 (0.6%) | 645 (0.5%) | 378 (0.9%) |
| Rheumatoid arthritis | 7,391 (1.5%) | 1,528 (1.9%) | 1,102 (1.8%) | 4,571 (1.6%) | 1,502 (1.2%) | 1,034 (2.4%) |
| ^1^Results are presented as median (IQR) ^2^Cancers excluding nonmelanoma skin cancer ^3^Mental and behavioural disorders due to psychoactive substance use  COPD: Chronic Obstructive Pulmonary Disease | | | | | | |

**Table 2.** **Gender-, age- and morbidity-characteristics for opioid users in 2017**

|  | Any opioid | Morphine | Oxycodone | Tramadol | Codeine | Other opioids |
| --- | --- | --- | --- | --- | --- | --- |
|  | (n=466,425) | (n=89,055) | (n=68,143) | (n=263,474) | (n=109,305) | (n=41,532) |
| Female sex (%) | 273,578 (58.7%) | 49,534 (55.6%) | 38,897 (57.1%) | 150,551 (57.1%) | 72,636 (66.5%) | 27,224 (65.5%) |
| Age (years)^1^ | 62 (48-74) | 66 (52-76) | 64 (50-75) | 60 (47-73) | 61 (48-72) | 74 (61-84) |
| Essential hypertension | 118,404 (25.4%) | 26,367 (29.6%) | 19,611 (28.8%) | 65,969 (25.0%) | 24,575 (22.5%) | 16,269 (39.2%) |
| Ischemic heart disease | 62,840 (13.5%) | 14,426 (16.2%) | 10,343 (15.2%) | 35,369 (13.4%) | 12,841 (11.7%) | 8,208 (19.8%) |
| Chronic kidney disease | 10,561 (2.3%) | 2,085 (2.3%) | 2,297 (3.4%) | 6,003 (2.3%) | 1,742 (1.6%) | 1,833 (4.4%) |
| Osteoarthritis | 111,078 (23.8%) | 23,843 (26.8%) | 22,824 (33.5%) | 61,675 (23.4%) | 22,280 (20.4%) | 12,565 (30.3%) |
| Diabetes | 65,357 (14.0%) | 14,225 (16.0%) | 10,147 (14.9%) | 37,498 (14.2%) | 13,894 (12.7%) | 7,449 (17.9%) |
| Affective disorders | 42,303 (9.1%) | 9,615 (10.8%) | 6,593 (9.7%) | 23,966 (9.1%) | 8,720 (8.0%) | 5,566 (13.4%) |
| Depression | 39,021 (8.4%) | 8,946 (10.0%) | 6,131 (9.0%) | 22,068 (8.4%) | 7,980 (7.3%) | 5,228 (12.6%) |
| Migraine | 10,391 (2.2%) | 1,778 (2.0%) | 1,510 (2.2%) | 5,650 (2.1%) | 3,263 (3.0%) | 850 (2.0%) |
| Cancer^2^ | 54,935 (11.8%) | 17,396 (19.5%) | 10,328 (15.2%) | 26,831 (10.2%) | 10,400 (9.5%) | 9,351 (22.5%) |
| Substance abuse^3^ | 44,723 (9.6%) | 10,802 (12.1%) | 7,098 (10.4%) | 26,036 (9.9%) | 8,606 (7.9%) | 4,707 (11.3%) |
| COPD | 37,444 (8.0%) | 10,589 (11.9%) | 6,163 (9.0%) | 20,313 (7.7%) | 7,417 (6.8%) | 5,561 (13.4%) |
| Pain | 57,421 (12.3%) | 15,686 (17.6%) | 12,027 (17.6%) | 28,652 (10.9%) | 9,098 (8.3%) | 10,691 (25.7%) |
| Acute | 5,207 (1.1%) | 1,355 (1.5%) | 1,016 (1.5%) | 2,796 (1.1%) | 963 (0.9%) | 809 (1.9%) |
| Chronic | 34,604 (7.4%) | 9,963 (11.2%) | 7,666 (11.2%) | 16,522 (6.3%) | 4,844 (4.4%) | 7,117 (17.1%) |
| Fibromyalgia | 2,975 (0.6%) | 552 (0.6%) | 450 (0.7%) | 1,773 (0.7%) | 645 (0.6%) | 390 (0.9%) |
| Rheumatoid arthritis | 7,392 (1.6%) | 1,654 (1.9%) | 1,265 (1.9%) | 4,315 (1.6%) | 1,444 (1.3%) | 979 (2.4%) |
| ^1^Results are presented as median (IQR) ^2^Cancers excluding nonmelanoma skin cancer ^3^Mental and behavioural disorders due to psychoactive substance use  COPD: Chronic Obstructive Pulmonary Disease | | | | | | |

**Table 3. Medical profile of prescribers issuing prescriptions of tramadol among prevalent tramadol users in 2016, 2017, 2018, and 2019**

| Type of prescriber | 2016 | 2017 | 2018 | 2019 |
| --- | --- | --- | --- | --- |
| General practitioner | 900,567 (94%) | 809,235 (94%) | 611,908 (93%) | 505,608 (92%) |
| Practicing specialist | 2,755 (0%) | 2,869 (0%) | 2,413 (0%) | 2,088 (0%) |
| Hospital doctor | 43,544 (5%) | 39,513 (5%) | 31,704 (5%) | 27,754 (5%) |
| Dentist | 884 (0%) | 777 (0%) | 616 (0%) | 670 (0%) |
| Other | 677 (0%) | 164 (0%) | 154 (0%) | 83 (0%) |
| Missing | 11,119 (1%) | 9,914 (1%) | 8,669 (1%) | 11,952 (2%) |

**Tabl****e 4. Medical profile of prescribers issuing prescriptions of tramadol among new tramadol users in 2016, 2017, 2018, and 2019**

| Type of prescriber | 2016 | 2017 | 2018 | 2019 |
| --- | --- | --- | --- | --- |
| General practitioner | 76,102 (68%) | 63,226 (66%) | 45,554 (64%) | 40,284 (62%) |
| Practicing specialist | 1,414 (1%) | 1,461 (2%) | 762 (1%) | 697 (1%) |
| Hospital doctor | 27,542 (25%) | 24,091 (25%) | 19,061 (27%) | 16,941 (26%) |
| Dentist | 1,319 (1%) | 1,262 (1%) | 1,057 (1%) | 1,317 (2%) |
| Other | 779 (1%) | 132 (0%) | 94 (0%) | 75 (0%) |
| Missing | 4,522 (4%) | 5,158 (5%) | 5,177 (7%) | 5,503 (8%) |

**Table 5. Number of unique prescribers among opioid users during 2016, 2017, 2018, and 2019**

| Number of prescribers | 2016 | 2017 | 2018 | 2019 |
| --- | --- | --- | --- | --- |
| Any opioid |  |  |  |  |
| 1 | 56.03% | 56.18% | 55.97% | 55.43% |
| 2 | 27.57% | 27.45% | 28.44% | 28.65% |
| 3 | 11.03% | 10.98% | 10.88% | 11.20% |
| 4 | 3.71% | 3.80% | 3.40% | 3.43% |
| 5 | 1.65% | 1.60% | 1.31% | 1.29% |
| Codeine |  |  |  |  |
| 1 | 73.63% | 73.60% | 74.37% | 75.49% |
| 2 | 19.58% | 19.55% | 19.99% | 18.63% |
| 3 | 4.94% | 5.02% | 4.41% | 4.65% |
| 4 | 1.38% | 1.31% | 0.91% | 0.94% |
| 5 | 0.47% | 0.52% | 0.32% | 0.29% |
| Tramadol |  |  |  |  |
| 1 | 67.17% | 69.03% | 70.78% | 71.76% |
| 2 | 23.57% | 22.49% | 21.94% | 21.64% |
| 3 | 6.77% | 6.22% | 5.64% | 5.20% |
| 4 | 1.75% | 1.66% | 1.28% | 1.11% |
| 5 | 0.74% | 0.60% | 0.36% | 0.29% |
| Oxycodone |  |  |  |  |
| 1 | 47.44% | 47.16% | 45.78% | 45.56% |
| 2 | 33.42% | 33.28% | 35.63% | 34.94% |
| 3 | 13.21% | 13.34% | 13.08% | 14.09% |
| 4 | 3.97% | 4.31% | 4.16% | 4.01% |
| 5 | 1.96% | 1.90% | 1.36% | 1.39% |
| Morphine |  |  |  |  |
| 1 | 52.89% | 52.58% | 53.80% | 53.24% |
| 2 | 29.56% | 30.27% | 30.23% | 30.08% |
| 3 | 12.29% | 12.22% | 11.68% | 12.07% |
| 4 | 3.85% | 3.54% | 3.18% | 3.50% |
| 5 | 1.41% | 1.39% | 1.12% | 1.11% |
